# Modeling Photoassociative Spectra of Ultracold NaK + K

**DOI:** 10.1021/acs.jpca.3c01823

**Published:** 2023-09-18

**Authors:** Baraa Shammout, Leon Karpa, Silke Ospelkaus, Eberhard Tiemann, Olivier Dulieu

**Affiliations:** † Institut für Quantenoptik, Leibniz Universität Hannover, Hannover 30167, Germany; ‡ Université Paris-Saclay, CNRS, Laboratoire Aimé Cotton, Orsay 91400, France

## Abstract

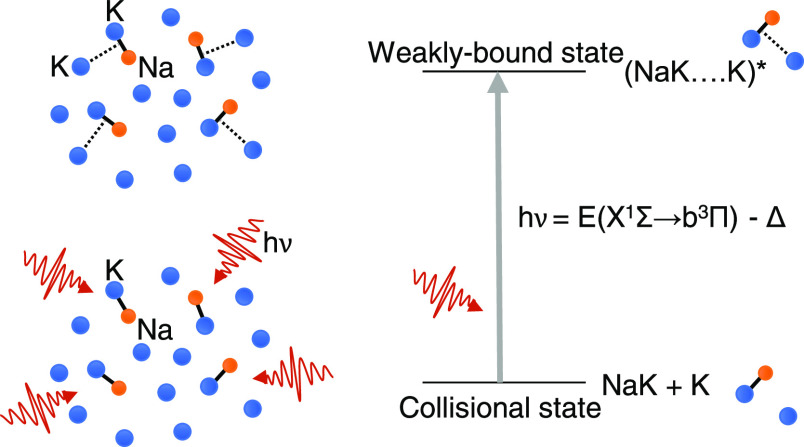

A model for photoassociation of ultracold atoms and molecules is presented and applied to the case of ^39^K and ^23^Na^39^K bosonic particles. The model relies on the assumption that photoassociation is dominated by long-range atom-molecule interactions well outside the chemical bond region. The frequency of the photoassociation laser is chosen close to a bound–bound rovibronic transition from the *X*^1^Σ^+^ ground state toward the metastable *b*^3^Π lowest excited state of ^23^Na^39^K, allowing us to neglect any other excitation, which could hinder the photoassociation detection. The energy level structure of the long-range ^39^K···^23^Na^39^K excited super-dimer is computed in the space-fixed frame by solving coupled-channel equations, involving the coupling between the ^23^Na^39^K internal rotation and the mechanical rotation of the super-dimer complex. A quite rich structure is obtained, and the corresponding photoassociation rates are presented. Other possible photoassociation transitions are discussed in the context of the proposed model.

## 1 Introduction

Photoassociation (PA) of particles A and B (which could be either atoms or molecules) in a dilute gas is a light-induced process, leading to the creation of a molecular complex AB by absorption of a photon with energy *h*ν: A + B + *h*ν → AB*, where *h* is the Planck constant, and ν is the photon’s frequency. In most cases, the AB complex is left in an excited state (thus the star symbol) due to the energy deposited by the photon. PA is a powerful way to induce unimolecular reactions, being the inverse process of photodissociation, both pertaining to the so-called half-collision, as elegantly discussed in ref (1). A sufficiently monochromatic light source can indeed populate a well-defined quantum state of the AB* complex. But a limitation immediately occurs at room temperatures: the broad width of the kinetic energy distribution of the particles, covering many bound levels of the complex, drastically hinders the possibility of preparing a well-defined quantum state of AB*.^2,3^

The ground-breaking development of laser cooling of atoms for more than forty years immediately appeared as an exquisite opportunity to use PA as a tool to study ultracold gases composed of alkali-metal atoms.^4−6^ The kinetic energy distribution of ultracold atoms is now narrower than most energy level spacings of the cold atom pair, which can efficiently absorb a photon to populate a molecular bound level (free-bound transition) in a quasi-resonant way, similar to a bound–bound transition. PA soon became an important high-resolution molecular spectroscopy technique: it allowed the population of weakly bound molecular levels^7,8^ with large spatial extension, as the atoms in an ultracold gas spend most of their time at distances much larger than the usual chemical bonds. PA spectroscopy thus advantageously complemented the few attempts to reach such levels via conventional molecular spectroscopy.^9^ Moreover, PA was the first approach to create samples of ultracold ground-state molecules,^10−12^ well before the method based on magnetoassociation, which also leads to the formation of ultracold molecules in selected individual quantum states.^11−13^

The opportunity to study atom-molecule collisions in the ultracold regime is a natural extension to atom–atom studies. Several experiments clearly observed losses in trapped molecular samples induced by the presence of atoms,^14−19^ which are presumably induced by atom-molecule scattering resonances.^20−23^ There is a vast literature about the modeling of atom-molecule collisions in the cold or ultracold regime. Focusing our interest on collisions involving ultracold alkali-metal atoms, which are quite heavy, open-shell, and with a strong electron-nuclear spin coupling, theorists suggested that such atom-molecule systems could be governed by a large number of scattering resonances due to the large amount of available rovibrational states of the diatom, requiring their statistical treatment.^24,25^ Resonances have been observed in various experiments.^20,22,26,27^ Triatomic NaK_2_ molecules have even been stabilized via such resonances.^28,29^ The model of refs (24) and (25) relies on the separation of the dynamics at large distances treated via quantum scattering theory from the dynamics at short distances treated via a formalism involving random matrix theory. Alternate models attempting to represent interactions and scattering resonances in a refined way have been proposed.^30−34^

The purpose of the present paper is to explore under which conditions PA could be an efficient approach (as suggested in an earlier paper^35^) to study mixtures of ultracold atoms and diatomic molecules as a natural extension of atom–atom PA and to create stable ultracold triatomic molecules. PA could also provide information about collisional dynamics by populating well-defined quantum levels just below an excited dissociation threshold A + B*. From a classical point of view, these weakly bound levels are excited at large interparticle distances, so that the motion starts inward with almost vanishing local kinetic energy. Similarly, an ultracold collision starts with very small kinetic energy at infinity. In both situations, i.e., colliding free particles or weakly bound particles, the system is sensitive to the strong short-range “chemical” interactions in the same manner. These interactions could thus be studied with PA as a function of the “initial energy”, i.e., the binding energies of the weakly bound levels, which could span a larger range than the ones that can be reached in a full collision. In the latter case, the investigation of the dynamics as a function of the initial kinetic energy is often not feasible due to constraints imposed by the experimental setup. Instead, magnetic Feshbach resonances^36,37^ are employed. These resonances occur when an external magnetic field causes a shift in the energy of weakly bound levels of the particle pair, aligning them with the energy of the initial state and creating a resonance with the initial kinetic energy.

**Figure 1:**
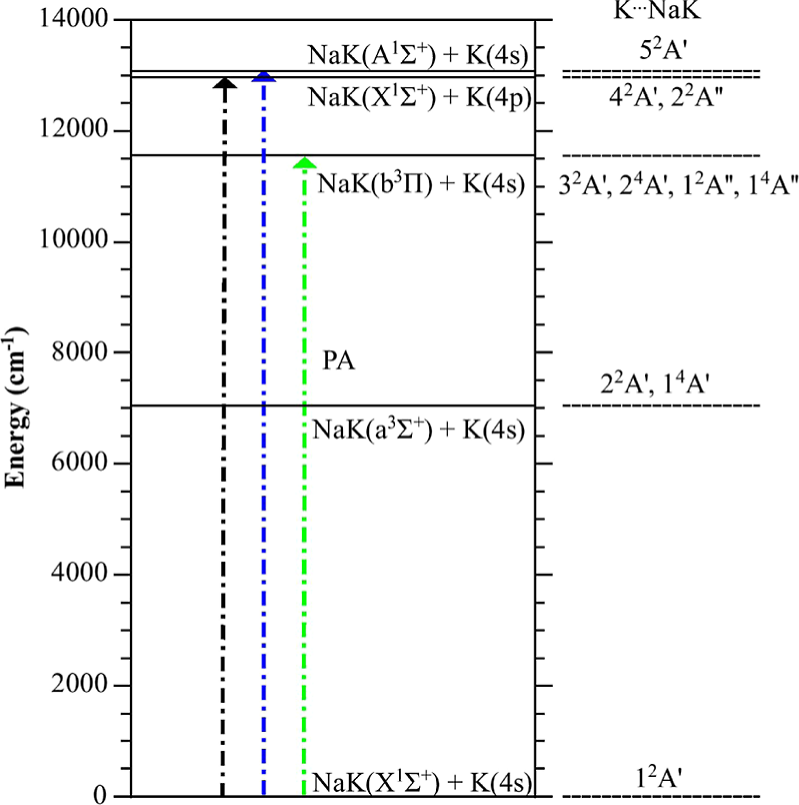
Simplified diagram of the energy levels of a K(4s) or K(4p) atom, combined with a NaK molecule in the lowest electronic states *X*^1^Σ^+^, *a*^3^Σ^+^, *b*^3^Π, and *A*^1^Σ^+^. The origin of the NaK energies is taken at the bottom of the *X*^1^Σ^+^ PEC, *r*_e_(*X*) = 6.6 a.u., located very close to that of the *b*^3^Π PEC, but very different from that of the other PECs (see Table 2). The vertical arrows depict possible vertical transitions from the *X*^1^Σ^+^ state. In green, the proposed atom-molecule PA transition: it is clearly distinct from the transition which would allow for PA of K atoms (in black). An alternate PA transition (in blue) could concern the NaK(*A*^1^Σ^+^) + K(4s) limit, but its energy at the chosen distance is close to the excitation energy of the K atom. The K···NaK electronic states defined in the *C*_*s*_ symmetry group representation (Section 3.1) and correlated to the limits above are listed on the right. A more comprehensive correlation diagram is reported in the Supporting Information.^41^

The main step of our theoretical approach, as in refs (35) and (38), only considers explicitly the long-range interactions between the atom and the molecule, while the short-range interactions are modeled via a boundary condition at a distance where the electronic exchange interaction is still negligible, around the so-called LeRoy radius.^39^ This relies on an assumption similar to the one holding for ultracold atom–atom collisions, that the probability density of the system at short distances is negligible compared to the one at large distances. For more complex systems like atom-molecule and molecule–molecule, this is in contrast with the statistical model above.^24,25^ The hypothesis of the dominant role of long-range interactions in ultracold atom-molecule Feshbach resonances has been recently invoked for ground-state K–NaK collisions.^22^ To exemplify our approach, we consider ultracold ^23^Na^39^K ground-state molecules immersed in a cloud of ultracold ground-state ^39^K atoms, which has recently been experimentally realized.^40^Figure 1 shows some of the lowest energy levels of the K–NaK pair. In contrast to previous work,^35,38^ where PA is studied using laser frequencies slightly detuned to the red of an atomic transition (black arrow in Figure 1), we choose a PA laser frequency close to a molecular transition (green arrow in Figure 1), thus addressing a “clean” spectral range outside of that of the atom–atom PA.

In the present case, the PA laser frequency ν is chosen close to the molecular transition frequency between the lowest rovibrational level *v*_*X*_ = 0, *j*_*X*_ = 0 of a ^23^Na^39^K molecule in its electronic ground state *X*^1^Σ^+^ (which can be experimentally prepared in suitable ensembles with high phase space density^42^) and the lowest rovibrational level *v*_b_ = 0, *j*_b_ = 1 of the Ω = 0^+^ component of the lowest excited electronic state *b*^3^Π (where Ω refers to the projection of the ^23^Na^39^K total electronic angular momentum on the diatomic molecular axis). Thus, the search for PA signals will not be hindered by the presence of NaK transitions, as the *b*^3^Π state is the lowest of all excited electronic states, which can be reached from the ground state by an electric dipole transition. At large atom-molecule distances, the transition electric dipole moment (TEDM) of ^23^Na^39^K determines the strength of the PA transitions: the *b*^3^Π state is weakly coupled by spin–orbit interaction to the neighboring *A*^1^Σ^+^ excited state, making this transition dipole-allowed. A PA scheme relying on the *A*^1^Σ^+^ state could be more difficult to identify because of overlap with the spectrum of the *A*–*X* transition in NaK.

In Section 2, we present our approach to derive the long-range potential energy curves (PECs) of the K–NaK complex based on advanced quantum chemistry methods. The results are collected in Section 3.1, and the asymptotic model for the calculation of bound levels of the NaK–K complex in Section 3.2. Finally, the corresponding PA rates are shown in Section 3.3 in the context of future experimental investigations.

In the following, we will omit the isotope labels and simply invoke K and NaK instead of ^39^K and ^23^Na^39^K. Unless otherwise stated, distances will be given in atomic units 1 a.u. ≡ *a*_0_, with *a*_0_ the Bohr radius,^43^ and energies in cm^–1^, a convenient unit for spectroscopy, or in atomic units (a.u.) or Hartrees.^43^ The electric dipole moment expressed in a.u. corresponds to the conversion factor 1 a.u. = 2.54175 D.^43^

## 2 Methods

We present in this section the chosen approach for the electronic structure calculations of NaK and K–NaK.

**Figure 2:**
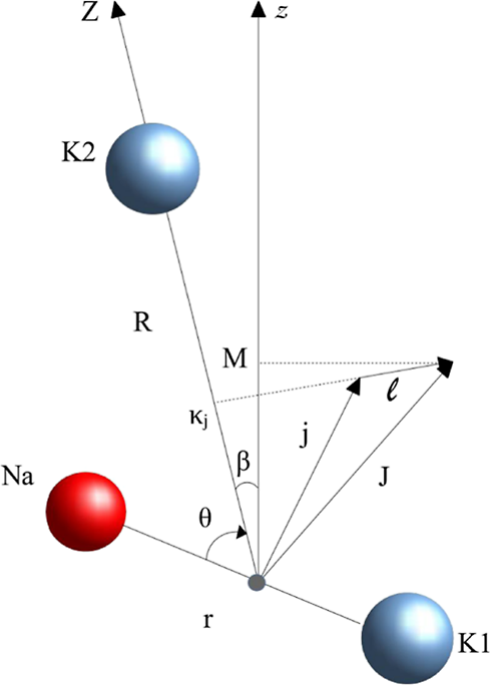
Chosen coordinates for the triatomic system NaK–K. The two K atoms are distinguished, which is consistent with a long-range approach. The Jacobi coordinates defined in the body-fixed (BF) frame (*XYZ*) are *R*, *r* and θ. The space-fixed (SF) frame (*xyz*) is characterized by the Euler angle β between the BF and SF axis *Z* and *z*. The total (electronic + rotation) angular momentum *j⃗* of NaK (with projection κ_*j*_ on the BF *Z*) is coupled to the mechanical rotation  of the NaK–K pair. In the absence of an external field, the resulting total angular momentum  is conserved, with a projection κ_*j*_ (resp. *M*) on the BF *Z* (resp. SF *z*) axis.

The K–NaK complex is described in Jacobi coordinates (Figure 2), *R* being the distance between the potassium atom K (noted K2) and the center of mass of the diatom NaK (with the K atom noted K1), *r* the bond length of NaK, and θ the angle between the vector *R⃗* pointing toward K2, and the diatomic axis pointing from K1 to Na. Thus, θ = 0 and θ = 180° correspond to the linear configurations K–NaK and NaK–K, respectively. We always assume that *R* ≫ *r*. At such a large distance *R*, the K–NaK interaction is significantly smaller than the energy separation (about 15 cm^–1^, extrapolated from the deperturbation analysis presented in Figure 8a of ref (44)) between the Ω = 0 and Ω = 1 spin–orbit components of the *b*^3^Π state: Ω = 0 is taken as a conserved quantum number, so that it is not necessary to consider the projection of the total electronic angular momentum of the diatom on the *Z* Jacobi axis. This approximation is sometimes identified as the super dimer model (see for instance ref (45), treating a similar situation).

We compute the electronic structure with the MOLPRO software package.^46,47^ The triatomic complex NaK–K is modeled as a three-valence-electron system, where the electrons of the atomic ion cores are replaced by large effective-core relativistic pseudopotentials (ECP)^48^ referenced as ECP18SDF for K^+^ and ECP10SDF for Na^+^. We use the valence basis sets associated with the ECPs in their uncontracted form, as implemented in MOLPRO. We added *spdf* diffuse functions with exponents reported in Table 1. In order to account for electronic correlations between the core and the valence electrons, we employ core polarization potentials (CPPs),^48^ parametrized by the electric dipole polarizabilities α of Na and K and cut-off radii ρ for each species (Table 1).

**Table 1:** For Each Species Na and K, Exponents for the *spdf* Diffuse Functions Completing the Basis Set Implemented in MOLPRO and Dipole Polarizabilities α and Cut-Off Radii ρ Defining the CPPs^48^

	exponents				CPP parameters in a.u.	
	*s*^a^	*p*^a^	*d*	*f*	α	ρ
Na	0.009202	0.005306	0.3, 0.07	0.09	0.9947	1.27
K	0.009433	0.004358	0.38, 0.04	0.04	5.354	1.86

a Adopted from ref (49).

The PECs of NaK and the potential energy surfaces (PESs) of the NaK–K complex are calculated for all distances using the multiconfiguration reference internally contracted configuration interaction (MRCI) method^50^ with Pople correction. The initial guess for orbitals is generated by the multi-configuration self-consistent field (MCSCF) method.^51^ The full PESs of the NaK–K complex will be used in a further publication, with the aim of contributing to the detailed understanding of the trimer structure in view of the most recent experimental work of ref (52). As stated in the Introduction, we present in the following sections a model for atom-molecule PA based on long-range interactions. It is well known that such quantum chemistry calculations have a limited numerical precision, which prevents them from accurately representing the long-range part of the PESs, as discussed, for instance, in refs (53) and (54) for similar bialkali systems. In the next section, we fit the long-range part of the PESs with a standard multipolar expression, and we argue that the overall behavior of the long-range PESs of the NaK–K complex is accurate enough to allow for a meaningful modeling of the PA process.

A good test of the appropriateness of the basis sets used at the MRCI level is given by comparing our results to other determinations of the PECs for the *X*^1^Σ^+^ and *b*^3^Π states of NaK. This is exemplified in Table 2 where the main spectroscopic constants of NaK are compared to the experimental ones, showing a satisfactory agreement of better than 1%. In the Supporting Information,^41^ we provide a direct comparison of the NaK PECs, which demonstrates satisfactory agreement over the entire PECs.

**Table 2:** Computed Spectroscopic Constants for the *X*^1^Σ^+^ and *b*^3^Π States of ^23^Na^39^K (This Work), Compared to Various Experimental Data: Equilibrium Bond Length *r*_e_, Potential Well Depth *D*_e_, Harmonic Constant ω_e_, Excitation Energy *T*_e_, and Rotational Constant *B*_e_^a^

		this work	exp.	refs
*X*^1^Σ^+^	*r*_e_ (a.u.)	6.58	6.612217(3)	(55)
	ω_e_ (cm^–1^)	123.27	124.013(8)	(55)
	*B*_e_ (cm^–1^)	0.096	0.09522934(1)	(55)
	*D*_e_ (cm^–1^)	5259	5273.62(10)	(56)
*b*^3^Π	*r*_e_ (a.u.)	6.60	6.62	(57)
	ω_e_ (cm^–1^)	120.21	120.407(4)	(57)
	*B*_e_ (cm^–1^)	0.096	0.09506(2)	(57)
	*T*_e_ (cm^–1^)	11558	11562.18	(57)
	*D*_e_ (cm^–1^)	6666	6697.9	(57)

a For completeness, we found the minimum of the *A*^1^Σ^+^ PEC located at *r*_e_ = 7.93 a.u.

## 3 Results

### 3.1 Long-Range PESs of K···NaK for the Excited States 3^2^*A*′ and 1^2^*A*″

At large distances, the weakly bound K···NaK complex (symbolized by the ··· symbol) has only two symmetry operations: the identity and the reflection through the mirror plane containing the vectors *R⃗* and *r⃗*. We describe it within the framework of the *C*_s_ point group, where the electronic wave functions are categorized into two irreducible representations, namely *A*′ and *A*″. These representations correspond to wave functions that are symmetric or antisymmetric under reflection through the mirror plane. The trimer’s spin multiplicity can be either a doublet or a quartet. The expected lowest electronic trimer states are depicted on the right side of Figure 1. The number in front of each symbol counts the states with equal symmetry from the bottom of the energy scale. We focus on the excited 3^2^*A*′ state that can be reached from the 1^2^*A*′ ground state by PA and on the 1^2^*A*″ state for completeness, which correlate to the asymptote NaK(*b*^3^Π) + K(4s) relevant for the chosen PA transition. Spin–orbit and hyperfine couplings are not introduced in the rest of the calculations. However, as stated in the introduction, the PA transition is allowed due to spin–orbit coupling between the *A*^1^Σ^+^ and *b*^3^Π states, giving rise to two states labeled as 0^+^. Thus, for the experimental implementation of the present model, the notation *b*^3^Π should actually be understood as the component *b*^3^Π (0^+^) of the triplet manifold. This leads to a reduction of the PA rate calculated in the next section by *a* factor of about two, expressing that only the A′ state will be involved in the PA process. For simplicity, in the following, this component is labeled with the *b* symbol only, while the *X*^1^Σ^+^, *a*^3^Σ^+^ and *A*^1^Σ^+^ are denoted with *X*, *a*, and *A*, respectively. The proposed experiment involves the lowest vibrational level *v*_b_ = 0 of NaK(*b*). The calculation mesh is defined in the following way: we vary the bond length *r* over the extension of the vibrational wave function of this *v* = 0 level by taking *n_r_= 9* values between 5.804 and 7.436 a.u. in steps of 0.204 a.u. A set of *n_θ_* = 19 values for θ between 0 and 180° with a 10° step size is adopted. We selected a variable grid step size δ*R* in *R* adapted to the variation of the long-range PESs, with *n_R_* = 29 values between 30 and 160 a.u. as follows: δ*R* = 2, 1, 5, 20 a.u., over the consecutive intervals [30*a*_0_–40*a*_0_], [40*a*_0_–50*a*_0_], [50*a*_0_–100*a*_0_], [100*a*_0_–160*a*_0_], respectively. In total, the three-dimensional long-range PESs are calculated on a mesh of *n_R_* × *n*_*r*_ × *n_θ_* = 29 × 9 × 19 = 4959 grid points.

In order to extract converged excited doublet states, we set the number of active orbitals to 6 (5 orbitals in *A*′ and 1 in *A*″ irreducible representations). We perform state-averaged MCSCF of the lowest five ^2^*A*′ states using configuration state functions. Subsequently, two distinct multireference CI calculations are achieved for the four ^2^*A*′ lowest states and for the lowest ^2^*A*″ state (including two states 1^2^*A*″ and 2^2^*A*″ in the internal CI for correct convergence).

**Figure 3:**
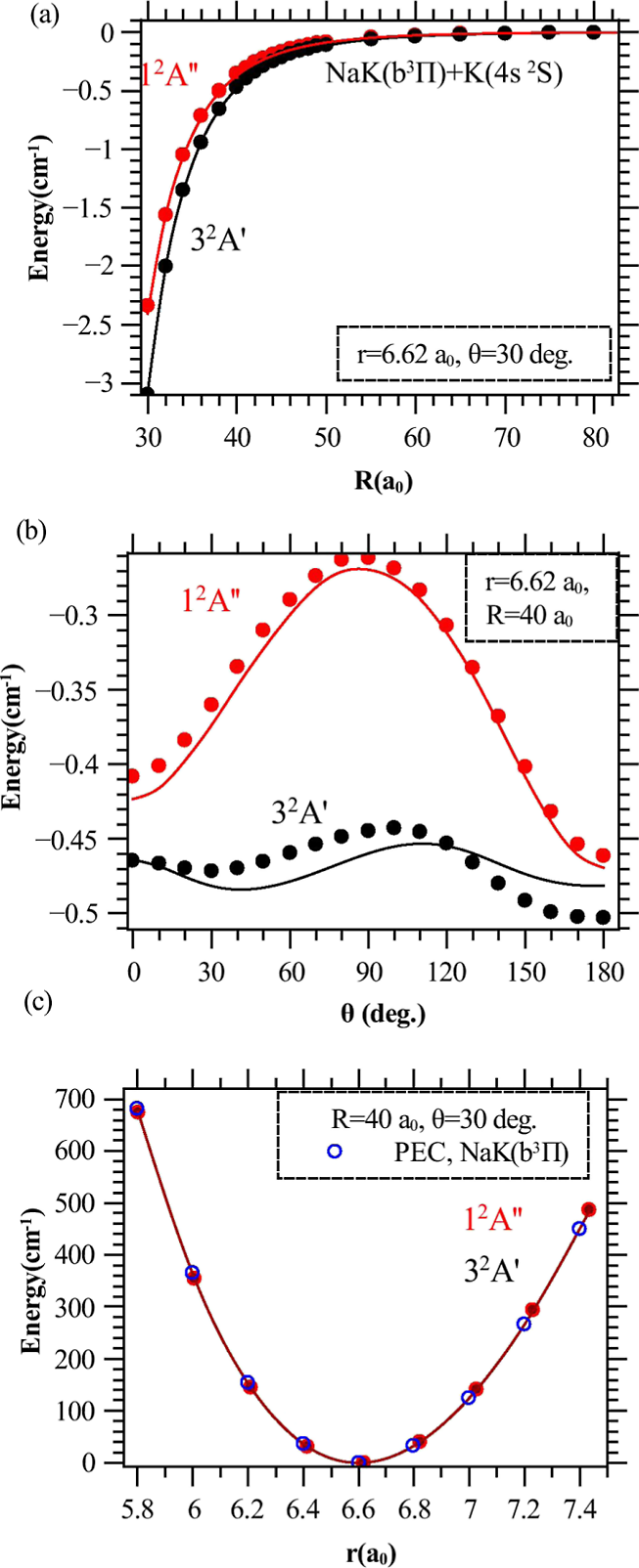
One-dimensional cuts through the long-range PESs of the 3^2^*A*′(black circles) and 1^2^*A*″ (red circles) states of K···NaK. The zero of energy is taken at the dissociation limit NaK(*b*^3^Π, *r* = 6.62 a.u.) + K(4s). Fits of the computed points according to eq 1 are displayed with solid lines. (a) At *r* = 6.62 a.u., θ = 30°. (b) At *r* = 6.62 a.u., *R* = 40 a.u. (c) At *R* = 40 a.u., θ = 30°, showing that the 3^2^*A*′ and 1^2^*A*″ PESs nicely match the NaK(*b*^3^Π) PEC (blue circles) over this *r* interval for such a large *R* (see Supporting Information).^41^

In Figure 3 we present three different cuts of the calculated long-range PES of the 3^2^*A*′ and 1^2^*A*″ states. The angular dependence of the PESs is exemplified in Figure 3b, at *r* = *r*_e_(*b*) = 6.62 a.u. and *R* = 40 a.u.: the anisotropy of the 1^2^*A*″ PES is more pronounced than the one of the 3^2^*A*′ PES. It is worth noticing that the 3^2^*A*′ and 1^2^*A*″ PESs should be degenerate in the linear geometry: the observed differences reflect the limited size of the chosen active space. These differences can be reduced by 1 order of magnitude by increasing the number of the active orbitals to 7 (five orbitals in *A*′ and 2 in *A*″ irreducible representations). However, such calculations are expensive, and as the overall structure of the PESs is not significantly changed, we keep the present results, which do not hinder a reliable estimate of the rates of the proposed PA scheme. For completeness, we present the two-dimensional long-range PES (in *R* and θ) for these two states in the Supporting Information.^41^

For the fixed geometry *r* = *r*_e_(*b*) = 6.62 a.u. and θ = 30°, the resulting cut of the 3^2^*A*′ PES is more attractive than the 1^2^*A*″ one for *R* > 30 a.u (Figure 3a). On the large energy scale of Figure 3c plotted for *R* = 40 a.u. and θ = 30°, both PES cuts look identical, approaching the *b* PEC of NaK.

The calculated long-range PES can be fitted to the standard multipolar expansion expressed in atomic units of distance and energy
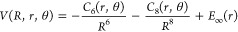
1where *E*_*∞*_(*r*) is the *r*-dependent energy of the K···NaK complex for *R* → ∞, thus identical to *b* PEC of NaK (see the Supporting Information).^41^ The dominant term characterized by the *C*_6_ van der Waals coefficient^58^ results from the cumulative effect of the Debye (or induction) interaction of a permanent dipole inducing an instantaneous dipole on a non-polar particle, and of the London (dispersion) interaction between the dipoles induced on each partner.^59−61^ The parameters in eq 1 are obtained via a two-step procedure: (i) by fitting *V*(*R*, *r*, θ) between *R* = 60 a.u. and *R* = 160 a.u. to the dominant term , and (ii) keeping *C*_6_(*r*, θ) and *E*(∞) fixed, and fitting *V*(*R*, *r*, θ) to eq 1 between *R* = 40 a.u. and *R* = 160 a.u. to estimate *C*_8_(*r*, θ). These fits describe the ab initio calculations to better than 1% at fixed θ (Figure 3a,c), while the fit of the angular dependence (Figure 3b) is slightly less satisfactory, with a deviation of about 2–4%.

**Figure 4:**
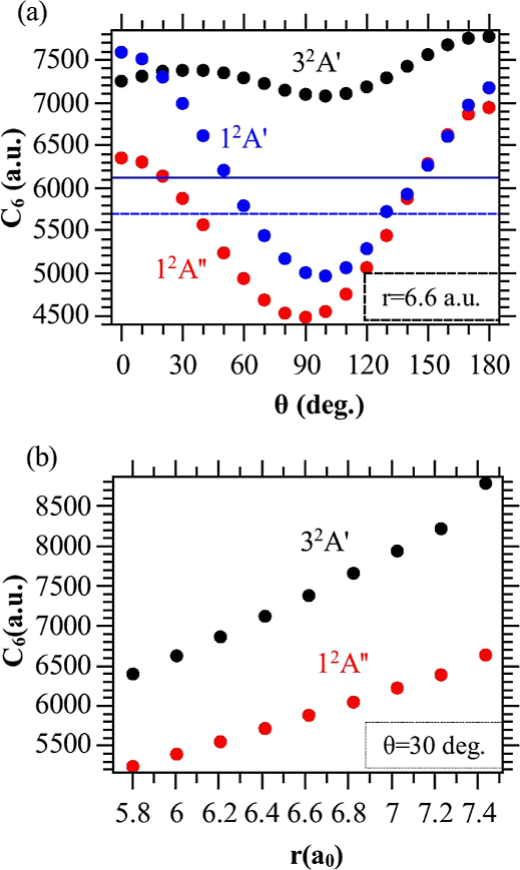
*C*_6_ coefficients of the long-range PES of the 1^2^*A*′ (blue circles) 3^2^*A*′ (black circles), 1^2^*A*″ (red circles) states of K···NaK, (a) as functions of θ at *r* = *r*_e_(*b*^3^Π) = 6.62 a.u. for 3^2^*A*′ and 1^2^*A*″ and at *r* = *r*_e_(*X*^1^Σ^+^) = 6.61 a.u. for 1^2^*A*′; (b) as functions of *r* at θ = 30°. In panel (a), the horizontal solid line gives the spherically averaged value *C*_6_(θ) for the ground state 1^2^*A*′ compared to the value of ref (62) (dashed line).

Figure 4 shows the fit results, which are also gathered in the Supporting Information.^41^ As expected, the anisotropy of the PESs is reflected in the variation of *C*_6_(*r* = 6.62 a.u., θ) (Figure 4a), decreasing by about 30% from θ = 0 to θ = 90° for the 1^2^*A*″ curve, and by about 5% from θ = 30 to θ = 90° for the 3^2^*A*′ curve. The resulting spherically averaged value  a.u. is found to be in reasonable agreement with the more accurate value of ref (62),  a.u., which is calculated from the individual properties of K and NaK. As anticipated in Section 2, such accuracy is sufficient for the determination of meaningful PA rates, which is the main goal of our study, without pretending to precisely determine the energy position of the PA levels of the NaK–K complex. Finally, the results in Figure 4b express the physics of the Debye interaction: the coefficient *C*_6_(*r*) (with θ = 30° in the figure) increases with *r*, as its dipole moment does in this region.^63^

Equation 1 is useful for easily calculating the long-range PESs at arbitrary values of *R*, *r* and θ, as this will be required for solving the Schrödinger equation for the atom-molecule relative motion in the next section.

### 3.2 Weakly Bound Energy Levels of the K···NaK Complex

We treat the K···NaK system in free space as an effective two-body problem (referred to as a super dimer model) in the space-fixed (SF)-frame *xyz*, assuming a total angular momentum *J⃗*. A ground-state K(4s^2^S) atom, considered structureless, approaching a diatomic molecule NaK in a given rovibrational level  with energy ε_*vj*_ of an electronic state  hinders the free rotation of the diatom, which generates anisotropy of the long-range K···NaK interaction potential *V*(*R*, *r*, θ), thus coupling the NaK rotational levels. The relevant angular momenta *j⃗*, , , are defined in Figure 2, with |*j* – | ≤ *J* ≤ *j* + . The corresponding operators will be denoted *Ĵ* , *ĵ*,and . In this section, we calculate the K···NaK weakly bound energy levels close to the dissociation limits K(4s) + NaK(*b*(*v*_b_ = 0, *j*_b_)) using a standard coupled-channel approach (see, e.g., ref (64)).

The Schrödinger equation *Ĥ*Ψ = *E*Ψ for the K···NaK system with eigenfunction Ψ and energy *E*, involves the Hamiltonian
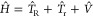
2with the kinetic energy operators associated with the *R* and *r* coordinates
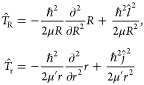
3and where μ = *m*(K)*m*(NaK)/[*m*(K) + *m*(NaK)] is the reduced mass of the complex K···NaK, and μ′ is the reduced mass of NaK. The operator *V̂* corresponds to the interaction potential *V*(*R*, *r*, θ) between K and NaK and includes the potential energy of the diatom, while the one of the isolated atoms is disregarded as a fixed quantity. As in the previous section, we keep the zero of energies as the energy of the K···NaK system for *R* → ∞ with , namely the location of the bottom of the PEC of the  electronic state (see the Supporting Information).^41^ In the following, the  index will be removed for simplicity.

We first define the basis set in the BF frame for a given *J* value with its projection *M*(65)
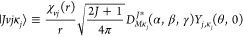
4Here χ_*v*,*j*_(*r*) is the rovibrational wave function with eigenvalue ε_*vj*_, and  is a spherical harmonic associated with the rovibrational level (*v*, *j*) of the  electronic state of the isolated NaK molecule. The projection of *j⃗* on the *Z* BF axis is denoted with κ_*j*_. The Wigner functions  refer to the transformation between the SF and BF frames and depend on the Euler angles (α, β, γ). The PES *V*(*R*, *r*, θ) can be recast in this basis set as a matrix with elements
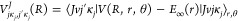
5where the notation ⟨∥⟩_*r*,θ_ denotes the integration over *r* and θ. The straightforward integration over Euler angles is performed but not labeled for simplicity, leading to diagonal terms in κ_*j*_ only. The diagonal term  represents the one-dimensional PECs of the super dimer. The off-diagonal elements  hold for the couplings between NaK rotational levels induced by the anisotropy of the interaction potential. As the energy range around the asymptote of K···NaK addressed in the rest of the paper is very small compared to the energy spacing of  vibrational levels, we restrict the basis set to *v* = 0, so that this index can be removed.

The BF basis set |*Jj*κ_*j*_⟩ is transformed to the basis set |*Jj*⟩ in the SF-frame according to^66−68^
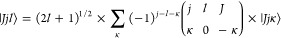
6where κ_*j*_ ≡ κ as *J⃗* and *j⃗* have the same projection on the *Z* axis, and the parentheses refers to 3*j*-Wigner symbols. The matrix elements  are transformed to the SF frame.
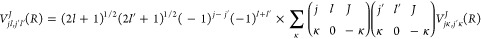
7In the SF frame, for a given *J*, the solution of the Schrödinger equation with an energy *E*_*n*_^*J*^ and total wave function |*J*;*E*_*n*_^*J*^⟩≡Ψ^*J*^(*R*;*E*_*n*_^*J*^) can be expanded as
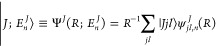
8where the radial channel wave functions  are solutions of the set of coupled equations
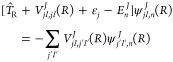
9
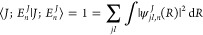
10where we define the partial norm as
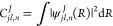
11and the rotational constant of the K···NaK super dimer as
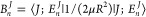
12

**Figure 5:**
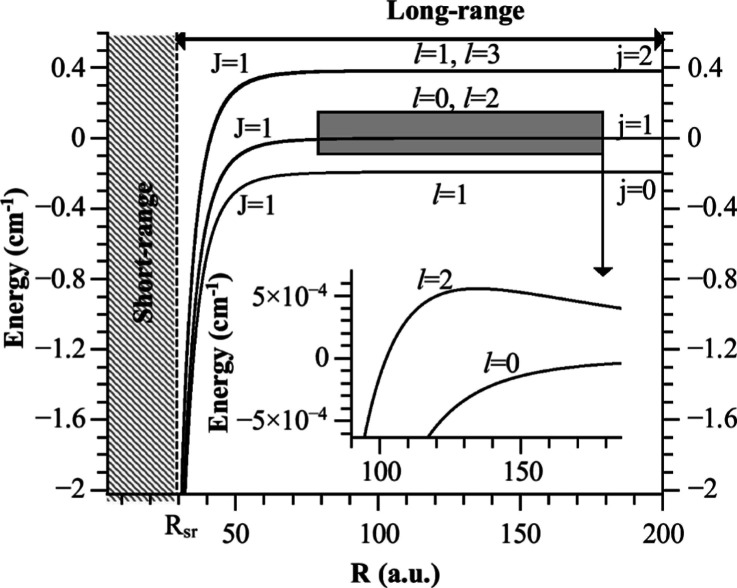
Effective long-range PECs of the NaK···K system in the SF frame for *J* = 1 as functions of *R*, *W*_*jl,jl*_^1^(*R*) = *V*_*jl,jl*_^1^(*R*) , involved in the coupled-channel calculations of the weakly bound states close to the NaK(*b*^3^Π, *v*_b_ = 0, *j*_b_) + K(4s) limit (eq 9). The couplings between the channels are ignored for atom-molecule separation *R* < *R*_SR_. The inset shows a magnified view of the gray box, with the contribution of the partial waves  = 0, 2.

The envisioned PA experiment starts from the 1^2^*A*′ state (Figure 1) with *J* = 0, *j* = 0,  = 0, and thus positive parity. We focus on the weakly bound energy levels of the 3^2^*A*′ and 1^2^*A*″ electronic states of the K···NaK complex, with *J* = 1 and negative parity . After convergence checks, we limit the number of coupled equations to *N*_max_ = 5 by including the five basis vectors |*Jjl*⟩ = |101⟩, |110⟩, |112⟩, |121⟩, |123⟩. The five diagonal elements *W*_*jl,jl*_^1^(*R*) = *V*_*jl,jl*_^1^(*R*)  in the Hamiltonian of eq 9, corresponding to these five channels, are displayed in Figure 5. This figure sets the range of energies where weakly bound states could be calculated for this system in the framework of our long-range hypothesis, namely in a window no larger than 2 cm^–1^.

The numerical solution of eq 9 requires the definition of the interaction potentials for *R* < *R*_sr_ (see Figure 5). We connect the diagonal terms  at *R*_sr_ = 30 a.u. to short-range Lennard-Jones potentials (expressed in a.u.) of the form
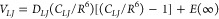
13for which the coefficients are gathered in the Supporting Information.^41^ Of course, the energy position of the computed weakly bound levels will depend on the whole PES and thus on the choice made for the short-range interactions. But the pattern of the energy spectrum, namely the density of levels, or their spacing, will overall be largely independent of the chosen parametrization for the short-range interactions. As an additional numerical simplification, the off-diagonal terms  are kept equal to their values at *R*_sr_ for *R* < *R*_sr_. In the Supporting Information,^41^ we illustrate that the eigenvalues of the system are insensitive to such a choice of the coupling terms.

We solve eq 9 with the mapped Fourier grid Hamiltonian (MFGH) method,^69^ considering a grid in *R* coordinate with 338 points, bounded by *R*_min_ = 5.6 a.u. and *R*_max_ = 1000 a.u. The origin of energies is taken at the NaK(*b*^3^Π, *v* = 0, *j* = 1) + K(4s) limit, associated with the dipole-allowed transition from the initial ground state system. Thus, all energy values relevant for the present study are negative. The computed energies of the weakly bound vibrational levels of the 3^2^*A*′ state, located below the NaK(*b*, *v*_b_ = 0, *j*_b_ = 0) + K(4s) limit, are presented in Table 3 (listed above the horizontal line), while those of the 1^2^*A*″ state are displayed in the Supporting Information.^41^ The reported lowest bound level at −2.08 cm^–1^ corresponds to an outer turning point of the PES at about 33 a.u., slightly outside the short-range region defined by *R*_sr_ = 30 a.u., such that the eigenvalue may already be influenced by the chosen PEC matching with the short-range region. As it was used in previous studies using MFGH, the rotational constant *B*_*n*_ reflects the spread in *R* of the probability density, which decreases with the binding energy, namely as the radial wave function extends toward large distances. Its variation is not smooth as it depends on the channels composing each eigenstate. This composition is analyzed with the partial norm . Between *n* = −15 and −1, 11 levels are dominated by a single channel, up to 90% or more, revealing that the couplings between the channels are weak in most cases. This is consistent with the weak anisotropy observed for the *A*′ state (Figure 3). In contrast, the more significant anisotropy of the *A*″ state is reflected in the strong channel mixing of the bound levels (see the Supporting Information.^41^) The partial norm *C*_10,*n*_^*J*^ in Table 3 is the only one to be considered for the present PA scheme, due to the dipole transition selection rules (see Section 3.3). Only five levels have a noticeable value of *C*_10,*n*_^*J*^ (larger than 0.2, labeled by a star), which could thus be expected to be detected in the experiment.

**Table 3:** Energies *E*_*n*_^*J*^ (with Respect to the NaK(*b*^3^Π, *v* = 0, *j* = 1) + K(4s) Limit), Rotational Constants *B*_*n*_^*J*^ of the Super Dimer (eq 12, Multiplied by 10^3^), and Partial Norms  (eq 11) of Weakly Bound Vibrational Levels (Numbered from the Uppermost One with a Negative Index, *n* = −15 to −1) of the 3^2^*A*′ (*J* = 1) State and Located Below the NaK(*b*^3^Π, *v* = 0, *j* = 0) + K(4s) Limit (at −0.19082 cm^–1^ on This Scale)^a^

*n*	*E*_*n*_^1^ (cm^–1^)	10^3^*B*_*n*_^1^ (cm^–1^)	*C*_01,*n*_^1^	*C*_10,*n*_^1^	*C*_12,*n*_^1^	*C*_21,*n*_^1^	*C*_23,*n*_^1^
–15	–2.08302	3.9359	**0.85860**	0.04450	0.09272	0.00161	0.00256
–14	–1.93277	3.9488	0.08529	0.00667	**0.88880**	0.01050	0.00874
–13	–1.91967	3.9441	0.05326	0.91628*	0.00132	0.01961	0.00953
–12	–1.64709	4.0032	0.00154	0.02128	0.00522	**0.96181**	0.01015
–11	–1.63070	4.0099	0.00159	0.01144	0.01209	0.00620	**0.96869**
–10	–1.22069	3.2778	**0.93890**	0.02159	0.03743	0.00086	0.00122
–9	–1.06302	3.2486	0.06009	0.28494*	0.65471	0.00001	0.00025
–8	–1.05143	3.2807	0.00028	0.67998*	0.29990	0.01230	0.00754
–7	–0.74335	3.2866	0.00338	0.01264	0.00332	**0.97989**	0.00077
–6	–0.72519	3.2785	0.01062	0.00323	0.00691	0.00107	**0.97817**
–5	–0.67031	2.5159	**0.94626**	0.02846	0.01150	0.00375	0.01003
–4	–0.50220	2.5616	0.02348	0.72385*	0.25078	0.00173	0.00017
–3	–0.48620	2.5563	0.00342	0.25827*	0.72796	0.00526	0.00509
–2	–0.35828	1.8134	**0.98749**	0.00426	0.00733	0.00040	0.00053
–1	–0.22396	1.0952	**0.97948**	0.00865	0.01037	0.00080	0.00070
1	–0.18263	0.5123	0.74792	0.22281	0.01269	0.01498	0.00161
2	–0.17464	0.8462	0.57744	0.00769	0.37116	0.02853	0.01519
3	–0.14644	2.1803	0.13784	0.06911	0.03322	0.74452	0.01531
4	–0.13015	2.2584	0.12186	0.02058	0.03386	0.00206	0.82164
5	–0.03704	0.9015	0.20546	0.70224	0.09101	0.00108	0.00021
6	–0.03150	0.6516	0.43635	0.10115	0.45990	0.00091	0.00168
7	–0.00033	0.3247	0.07172	0.92341	0.00467	0.00012	0.00007

a The stars label levels which could be seen in the proposed PA scheme. The same quantities are displayed for predissociating resonances (*n* = 1 to 7) located between the NaK(*b*^3^Π, *v* = 0, *j* = 0, 1) + K(4s) limits (see Supporting Information.^41^)

The MFGH method complemented with the stabilization method (see, for instance, ref (70)) allows for the localization of the quasibound levels, or predissociating resonances, located between the NaK(*b*, *v*_b_ = 0, *j*_b_ = 0, 1) + K(4s) limits and listed below the horizontal line in Table 3. Their characterization and the possibility of detecting them in the proposed PA scheme are discussed in the Supporting Information.^41^

### 3.3 PA Rate of K and NaK

We consider the initial state, denoted as *i*, to consist of a ground-state K atom and a ground-state NaK molecule. As they approach each other at a large separation distance *R*, they absorb a photon. This process leads to the formation of a weakly bound level of the electronically excited complex K···NaK in a final state denoted as *f* (eq 8). Under typical experimental conditions,^40^ the number of ultracold NaK molecules in the ultracold sample is much smaller than the number of atoms, so we define the number density of the minority particles as *n*_NaK_. In this PA process, the photon energy *h*ν_PA_ = *hc*/λ_PA_ is assumed to be slightly smaller than that of a NaK electronic transition, which will be specified below. Following the above sections, the diatom actually absorbs the photon while it is perturbed by the atom, so that the TEDM  of NaK characterizes the strength of the chosen PA transition. Therefore, the PA rate *R*_*if*_, i.e., the number of K···NaK electronically excited complexes per unit time and per diatomic molecule can be calculated in a similar way as for atom–atom PA.^35,38,71,72^ Its expression in SI units (s^–1^) for an ultracold atom-molecule sample at temperature *T* exposed to a PA laser with intensity *I*_PA_ and Boltzmann-averaged is
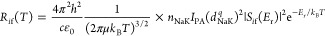
14involving the Planck constant *h*, the speed of light *c*, the Boltzmann constant *k*_B_, and the vacuum permittivity ε_0_. As above, μ refers to the reduced mass of the K···NaK complex. The energy *E*_r_ is the atom-molecule collision energy, which satisfies the resonance condition for the PA transition. The *q* cartesian component of  characterizes the active transition in the NaK diatom, namely the *X* → *A* electronic transition in the present case. The squared integral
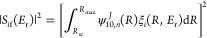
15expresses the spatial overlap (restricted to the long-range region relevant for the present study) between the continuum radial wave function ξ_*i*_(*R*, *E*_r_) of the K···NaK complex of the entrance channel, and the relevant radial component ψ_10,*n*_^*J*^(*R*) of the total wave function Ψ_f_(*R*, *E*_*n*_) of eq 8. According to ref (71), this rate is expected to vary as 1/*T*.

It is useful to derive the PA rate *K*_*if*_(*T*) normalized to the photon flux *I*_PA_λ_PA_/(*hc*) and to the molecular density *n*_NaK_ in SI units (m^5^) as
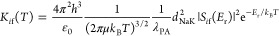
16to discuss the PA efficiency independently of particular experimental conditions.

In the initial state *i* of PA, we assume that the K(4s) atom collides in the *s*-wave regime ( = 0) with a NaK molecule in the (*v*_*X*_ = 0, *j*_*X*_ = 0) lowest rovibrational level of its electronic ground state *X*. It is thus represented by a single scattering channel with *J* = 0, which is identical in the BF and SF frames. Its PES results from the previous section, with a long-range *C*_6_^*X*^(θ,*r* = 6.6 a.u.) coefficient displayed in Figure 4a. As we disregard the short-range interactions above, we model the entrance channel with a single PEC of Lennard-Jones type in its original form^73^. The isotropic coefficient  a.u. is obtained by spherically averaging *C*_6_^*X*^(θ), and shows reasonable agreement with the value  a.u. of ref (62) obtained from dynamic polarizability calculations. The energy ε_d_ = 2201 cm^–1^ is of similar magnitude as the well depth of the NaK_2_ electronic ground state with respect to the energy of K(4s) + NaK(*X*, *r* = 6.6 a.u.) that we computed along the lines of Section 2. The resulting energy-normalized radial wave function ξ_*i*_(*R*, *E*_r_) is computed with the standard Numerov integration method between *R*_min_ and *R*_max_. Just like for the excited states above, the wave function ξ_*i*_(*R*, *E*_r_) and its associated scattering length obviously depend on the entire PEC. But in the context of the present long-range model, the amplitude and oscillation frequency of ξ_*i*_(*R*, *E*_r_) at large distance are the only relevant properties determining the pattern of the computed PA rate.

The transition dipole moment is taken from the calculations of ref (74), revealing that the *v*_b_ = 0 level of the *b* electronic state contains a fraction of ζ = 1.5 × 10^–4^ of the *A* electronic state, so that , where *d*_NaK_^*q*^(*X* → *A*) is the TEDM between the *X* NaK ground state and its *A* excited state coupled to the *b* state by spin–orbit interaction, as previously quoted (eq 15). As the bottoms of the *X* and *b* PECs have very similar shapes and an almost equal equilibrium distance (Table 2), the relevant vibrational wave functions of the dimer perfectly overlap, so that we chose *d*_NaK_^*q*^(*X* → *A*) = 3.818 a.u. at *r* = 6.6 a.u.^75^

**Figure 6:**
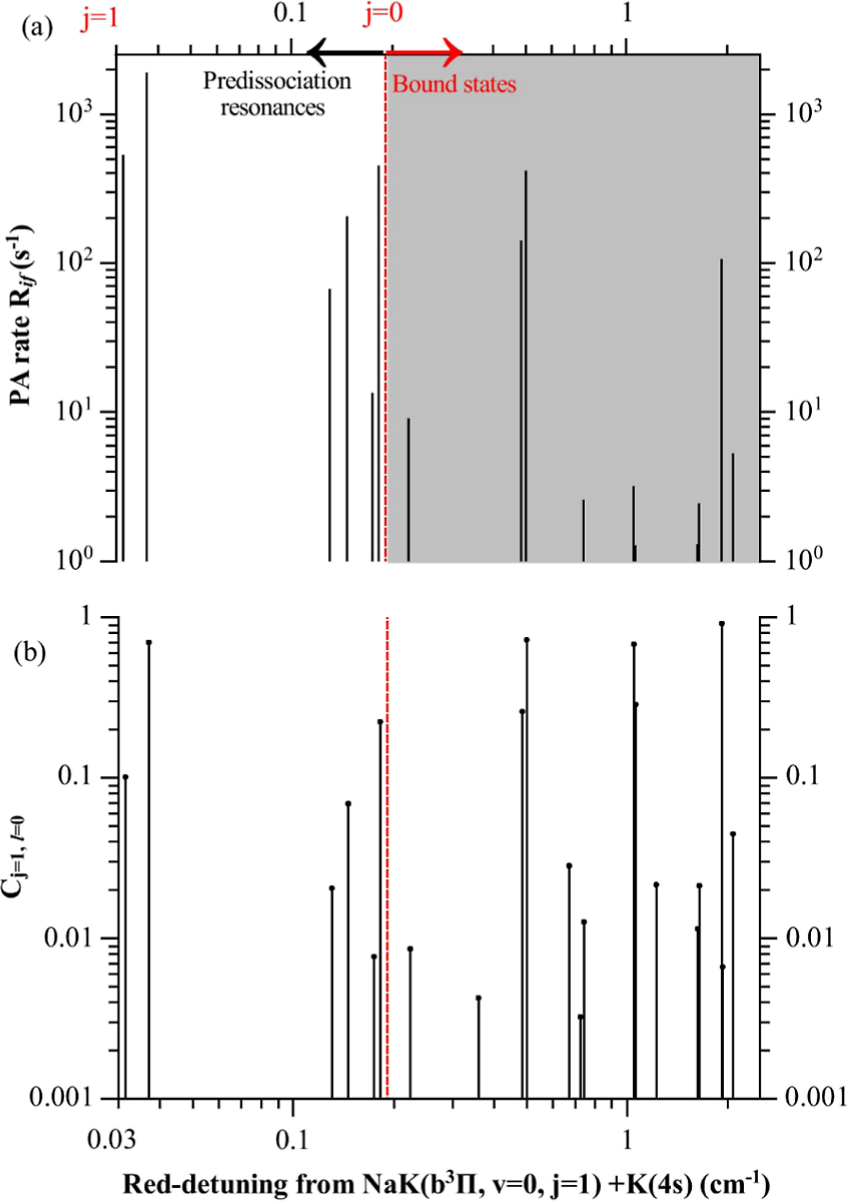
(a) PA rate *R*_*if*_(*T* = 200 nK) (eq 14) of NaK and K as a function of the red detuning (in cm^–1^, displayed as positive values due to the log scale) of the PA laser with respect to the transition *X*, *v*_*X*_ = 0, *j*_*X*_ = 0 → *b*, *v*_b_ = 0, *j*_b_ = 1, for a PA laser intensity *I*_PA_ = 100 W·cm^–2^ and a density of minority particles NaK of *n*_NaK_ = 10^11^ cm^–3^, representative of the experimental conditions.^40^ The energy position of the *b*, *v*_b_ = 0, *j*_b_ = 0 level (vertical red arrow) on this scale is 0.1908 cm^–1^. We computed bound levels (above this value, gray area) and predissociating resonances (below this value, white area). (b) Partial norm *C*_10,*n*_^1^ from Table 3, at the same energy scale.

**Figure 7:**
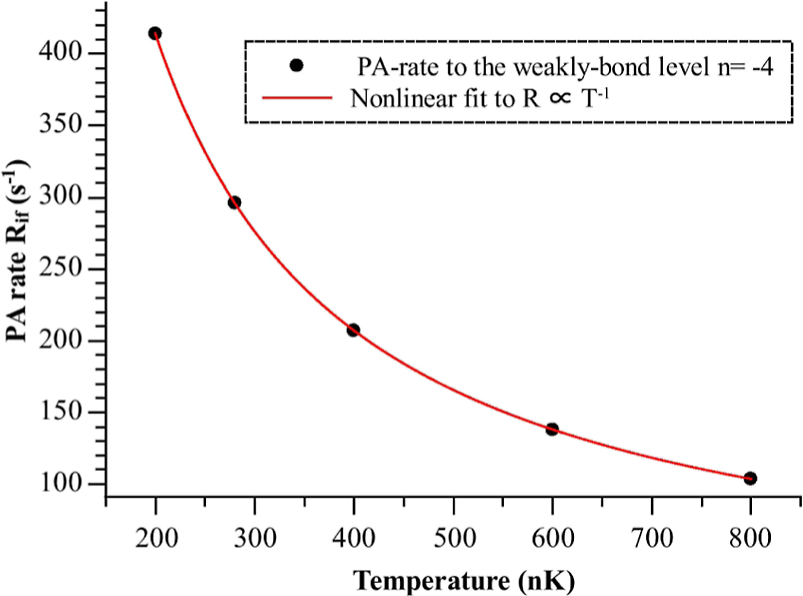
(a) PA rate *R*_*i*f_(*T*) of NaK and K as a function of the temperature with the same conditions as in Figure 6, and for the most intense line labeled as *n* = −4 in Table 3.

Due to the dipolar transition selection rules (Δ*J* = ±1, Δ*j* = ±1, Δ = 0), only the channel wave function component associated with |*J* = 1, *j* = 1,  = 0⟩ contributes to the squared integral (eq 15). The results for the PA rate (in s^–1^) for conditions relevant to the proposed experiment are displayed in Figure 6a. Assuming an average collision energy *E* = *k*_B_ × 200 nK between K and NaK, with a molecular density *n*_mol_ = 10^11^ cm^–3^ and a PA laser intensity *I*_PA_ = 100 W/cm^2^, the expected PA rate reaches up to several hundred events per second for three weakly bound levels. Figure 6b reports the values of the partial norm *C*_10,*n*_^1^ over the same energy range. The difference of patterns between the two panels, in particular the change of the relative amplitude of the results for the highest bars in panel (a), compared to panel (b), illustrates the constructive (for the *n* = −13, −4 levels) or destructive (for the *n* = −8 level) interference between the initial and final radial wave functions (see Supporting Information).^41^Figure 7 confirms that the PA rate indeed decreases as 1/*T* as T increases.

The normalized PA rate *K*_*i*f_ is at most on the order of 10^–30^ cm^5^, thus about 5 orders of magnitude smaller than the results obtained in cases where the PA transition is tuned to the atomic resonance.^35,38^ It is related to the squared ratio between the molecular TEDM, *d*_NaK_^*q*^(*X* → *b*) = ζ^1/2^*d*_NaK_^*q*^(*X* → *A*) ≈ 0.046 a.u., and the atomic one, the latter being 2 orders of magnitude larger than the molecular one. The expected density of PA resonances is lower in our case, as the long-range atom-diatom PECs vary as *R*^–6^, compared to the *R*^–5^ behavior of the quadrupole–dipole interaction induced by the quadrupole moment of the atomic ^2^*P* state.^35,38^

## 4 Concluding Remarks

We argue below that the proposed PA scheme is expected to induce a detectable signal under the conditions of our reference experiment.^40^ Our approach differs from the methods described in refs (35) and (38) in one significant aspect: we opt to tune the frequency of the PA laser in proximity to a molecular transition of NaK, rather than an atomic ^2^*S* → ^2^*P* transition. This choice is made to eliminate any PA lines associated with the formation of K_2_ dimers. By doing so, we aim to enhance the selectivity and sensitivity of our PA scheme, making it more suitable for our experimental setup. Moreover, the chosen NaK transition, *X*, *v*_*X*_ = 0, *j*_*X*_ = 0 → *b*^3^Π(0^+^), *v*_b_ = 0, *j*_b_ = 1, reaches the vicinity of the lowest possible excited rovibrational level of NaK, such that there is only a single open dissociation channel nearby for the photoassociated trimer, namely, K(4s^2^S) + NaK(*b*^3^Π(0^+^), *v*_b_ = 0, *j*_b_ = 0). Due to our hypothesis of dominant long-range interactions, it is unlikely that the hyperfine structure would play a significant role in the PA process. Indeed, both the initial and final states involve NaK molecules with a projection Ω = 0 of the total electronic angular momentum, inducing hyperfine splittings with a magnitude of a few tens of kHz (see, for instance, comparable discussions of Rb_2_,^76^ or KCs^77^).

The PA signal will result from the loss of the weakly bound photoassociated trimers from the optical trap, following their subsequent spontaneous emission within typically a few tens of ns. This loss signal will emerge from a background signal free from other diatomic resonant processes, as stated above. The predicted PA rate of about 100 s^–1^ is larger than the typical loss rate of the ground-state NaK molecules from the trap, 10 s^–1^, and thus fast enough to induce a detectable signal. If the photoassociated atom-molecule bound level is dominated by long-range interactions, as assumed in the present model, narrow PA lines should then be recorded. These lines may be slightly broadened by the possible predissociation toward the neighboring K(4s^2^S) + NaK(*b*^3^Π(0^+^), *v*_b_ = 0, *j*_b_ = 0) channel, as it has already been reported for potassium–potassium PA.^78^ It could also happen that the PA laser addresses weakly bound levels which are actually strongly dominated by short-range interaction, which thus would not appear anymore as narrow isolated lines but instead as a broad profile containing many closely spaced resonances. They would contribute to an increase of the molecule decay rate in an apparently non-resonant manner. Overall, we anticipate that PA signals from the atom-molecule system will be observable when starting from a molecular transition. The resulting structure of the signals should not be overly dense, allowing for a meaningful interpretation.

An alternate PA scheme is to tune the PA laser close to a dipole-allowed transition of NaK, such as *X*(*v* = 0, *j* = 0) → *A*(*v* = 0, *j* = 1), or *X*(*v* = 0, *j* = 0) → *B*^1^Π(*v* = 0, *j* = 0) (labeled as *B* in the following). However, while the corresponding TEDM *d*_NaK_^*q*^(*X* → *A*)(*r*) and *d*_NaK_^*q*^(*X* → *B*)(*r*) are sizable,^75^ the minima of the *A* and *B* PECs are not aligned with the one of the *X* PEC, leading to a small overlap of the corresponding diatomic vibrational wave functions and thus reducing the transition dipole moment (see eq 15). These values would presumably lead to PA rates of comparable magnitude to those of the present PA scheme. A full modeling of these options will be treated in a forthcoming work. Moreover, numerous predissociation channels would then be opened for the photoassociated weakly bound trimers, which could broaden the PA lines in a noticeable way.

Instead of looking for a trap-loss signal to probe PA, one option, inspired by the experimental demonstration of ref (79), could be to use a UV laser pulse to ionize the photoassociated weakly bound trimers, resulting in easily detectable diatomic or triatomic ions. As in the pioneering experiment of PA of cesium atoms,^10^ a sufficiently long-time delay between the PA laser and the ionizing laser pulse could also probe the formation of ultracold ground-state NaK_2_ trimers, created after the spontaneous decay of the photoassociated weakly bound trimers.

As we were correcting the present paper, the first experimental observation of atom-molecule PA was reported:^52^ the authors focused on the PA of deeply bound levels of the NaK_2_ excited electronic states, far more detuned (by about 5100 cm^–1^) than in our present work. As explained above, this corresponds to levels lying in the short-range region of the corresponding PESs, which are unknown and thus beyond the applicability of the present model.

## Supporting Information Available

Additional results on electronic structure calculations for the diatom NaK and the complex K···NaK; parameters of the Lennard-Jones potentials used to extend the diagonal elements of the effective potentials in the short-range; computed bound and quasibound levels of the 1^2^*A*″ state; examples of radial wave functions of the K···NaK complex; calculated predissociating resonances (PDF); and obtained *C*_6_ sand *C*_8_ coefficients from fitting the long-range PESs of the 3^2^*A*′ and 1^2^*A*″ states of K–NaK (PDF)
