# Supplementary material for: Modeling Photoassociative Spectra of Ultracold NaK + K
Source: J Phys Chem A. 2023 Sep 18;127(38):7872–83. doi: 10.1021/acs.jpca.3c01823 (PMC10544012; doi:10.1021/acs.jpca.3c01823)
Supplement: Supplementary file 1 — jp3c01823_si_001.pdf [file jp3c01823_si_001.pdf]

Supporting Information
Modeling Photoassociative Spectra of Ultracold NaK+K

Baraa Shammout*,[1] Leon Karpa,[1] Silke Ospelkaus,[1] Eberhard Tiemann,[1] and Olivier Dulieu*[2]
[1)] *Institut für Quantenoptik, Leibniz Universität Hannover, 30167 Hannover, Germany*
[2)] *Université Paris-Saclay, CNRS, Laboratoire Aimé Cotton, Orsay, 91400, France*

(*Electronic mail: olivier.dulieu@universite-paris-saclay.fr)

(*Electronic mail: shammout@iqo.uni-hannover.de)

(Dated: 20 August 2023)

I. ADDITIONAL RESULTS ON ELECTRONIC STRUCTURE CALCULATIONS

In Fig. S1 we compare our calculated PECs for the X and b electronic states of NaK with the results obtained with the CIPSI approach (Configuration interaction by perturbation of a multiconfiguration wave function selected iteratively), with large ECP and additional atomic-state-dependent CPPs[1,2]. The overall agreement is very satisfactory, as it was anticipated from Table II of the main text.

Figure S2 displays the two-dimensional PESs of the $3^2A'$ (in black) and $1^2A''$ (in red) at $r = r_e(b) = 6.62$ a.u., where the isopotential lines reflect the different anisotropy of the two PESs at large distances, as noticed in Fig. 3 of the main text. A more detailed presentation of the full PESs will be presented in a forthcoming paper.

In Fig. 3c of the main text, we already invoked, that our calculations of the $3^2A'$ PES nicely converge for large R toward the b PEC of NaK, over a restricted range of r values. We actually explored further this check of the accuracy of the present MRCI computations by calculating the PESs of K$\cdots$NaK at $R = 100$ a.u., where the NaK PECs should be reproduced independently of the angle θ. We extended our computations by increasing the number of active orbitals to 7 (*i. e.* 5 (2) states for the A' (A'') irreducible representations, respectively), and we performed the state-averaged MCSCF of the lowest seven $^2A'$ states and the lowest three $^2A''$ states. We achieved a good convergence of the five lowest $^2A'$ PESs, and of the two lowest $^2A''$ PESs, toward the lowest NaK PECs. Figure S3 thus presents an extension of both Figs. 1 and 3c of the main text, resulting in a correlation diagram. Due to the avoided crossing between the $3^2A'$ and $4^2A'$ states at $r = 7.55$ a.u. resulting from the known crossing between the b and A PECs, the correlation of the $3^2A'$ switches from the b state (as assumed in the present paper) to the A state beyond this distance. As anticipated in Fig. 1 of the main text, the $4^2A'$ and $5^2A'$ PESs undergo an avoided crossing at $r = 6.67$ a.u., close to the equilibrium distance of the X and b PECs of NaK. It corresponds to the crossing between the PA channel where K is excited, and the one where NaK is excited in the A state.

II. LENNARD-JONES POTENTIALS AT SHORT-RANGE

Table S1 lists the parameters of the Lennard-Jones potentials used to extend the diagonal elements of the effective potentials $W^J_{j\ell,j\ell}(R) = V^J_{j\ell,j\ell}(R) + \varepsilon_j + \ell(\ell+1)/(2\mu R^2)$ in Eq. 9 of the main text, below the distance $R_{\rm sr} = 30$ a.u.. The parameter $D_{LJ} = 0.15$ a.u. is chosen equal for all channels, leading to a sufficiently deep potential to minimize the probability density at this short range, and thus its influence on the eigenvalues.

TABLE S1: Parameters of the Lennard-Jones potentials (Eq. 13) extending below $R_{\rm sr} = 30$ a.u. the diagonal elements $W^J_{j\ell,j\ell}(R)$. The energies $E(\infty)$ correspond to the location of the $j = 0,1,2$ of NaK.

Diagonal term $W_{jl,j'l'}$	C_{LJ} (a.u.)	$E(\infty)$ a.u.
$W^1_{01,01}$	43685	0
$W^1_{10,10}$	43809	8.702×10^{-7}
$W^1_{12,12}$	46704	8.702×10^{-7}
$W^1_{21,21}$	46949	2.610×10^{-6}
$W^1_{23,23}$	46940	2.610×10^{-6}

III. COMPUTED BOUND AND QUASIBOUND LEVELS OF THE $1^2A''$ STATE

We display in Table S2 the energies E^J_n (with respect to the NaK(b, $v_b = 0, j_b = 1$)+K($4s$)) limit, the rotational constants B^J_n multiplied by 10^3 (Eq. 12 of the main text), and the partial norms $C^J_{j\ell,n}$ (Eq. 11 of the main text) of the weakly-bound vibrational levels (numbered from the uppermost one with a negative index, $n = -13$ to -1) of the $1^2A''$ ($J = 1$) state, and located below the NaK(b, $v_b = 0, j_b = 0$)+K($4s$) limit (at -0.19082 cm^{-1} on this scale). We see from the partial norms that the corresponding channel functions are more mixed than for the levels of A' symmetry, due to the more significant anisotropy of the A'' PES (Fig. 3 of the main text) compared to the A' one.

The same quantities are displayed for predissociating resonances (n =1 to 5) located between the NaK(b, $v_b = 0, j_b = 0,1$)+K($4s$) limits.

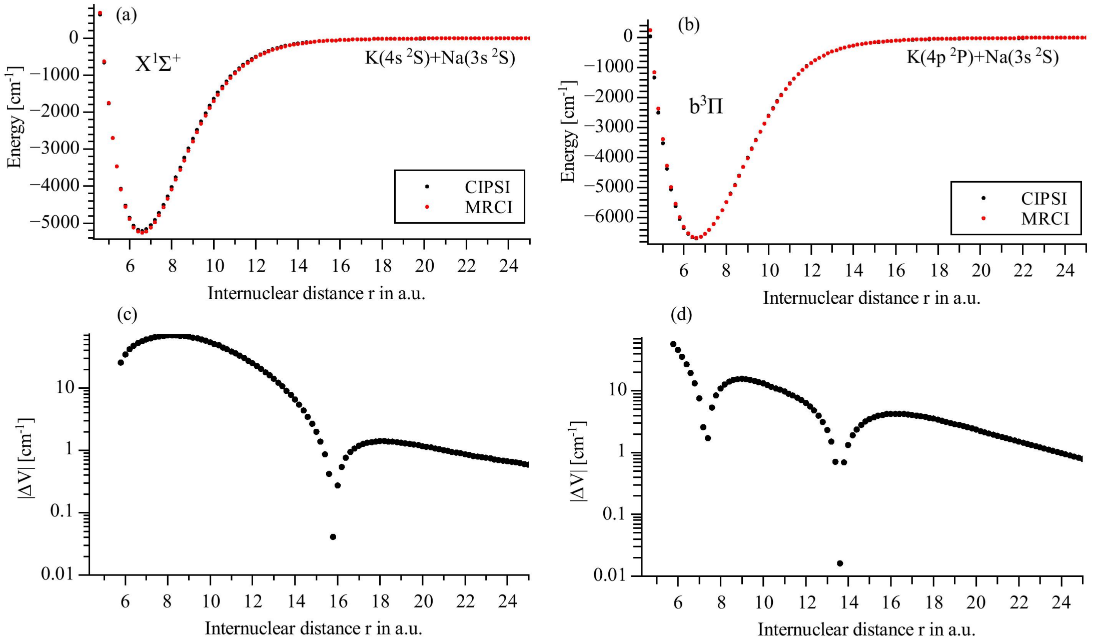

FIG. S1: Computed PECs for (a) the $X^1\Sigma^+$ ground state and (b) the $b^3\Pi$ excited state of NaK from the present work (MRCI, red dots), and from Refs.1 and 2 (CIPSI, black dots), and the energy difference $\Delta V = V_{\mathrm{MRCI}} - V_{\mathrm{CIPSI}}$ for (c) $X^1\Sigma^+$, and (d) $b^3\Pi$, respectively. The zero of energy is taken at the corresponding asymptotes Na(3s)+K(4s) and Na(3s)+K(4p), respectively.

TABLE S2: Energies E_n^J (with respect to the NaK($b^3\Pi$, $v=0, j=1$)+K(4s) limit), rotational constants B_n^J multiplied by 10^3 of the super dimer (Eq. 12 of the main text), and partial norms $C^J_{j\ell,n}$ (Eq. 11 of the main text) of weakly-bound vibrational levels (numbered from the uppermost one with a negative index, $n=-13$ to -1) of the $1^2A''$ ($J=1$) state, and located below the NaK($b^3\Pi$, $v=0, j=0$)+K(4s) limit (at -0.19082 cm^{-1} on this scale). The stars label levels which could be seen in the proposed PA scheme. The same quantities are displayed for predissociating resonances (n =1 to 5) located between the NaK($b^3\Pi$, $v=0, j=0,1$)+K(4s) limits

n	E_n^1 (cm^{-1})	$10^3 \times B_n^1$(cm^{-1})	$C^1_{01,n}$	$C^1_{10,n}$	$C^1_{12,n}$	$C^1_{21,n}$	$C^1_{23,n}$
-13	-1.61635	4.4511	0.17597	0.00018	0.00014	0.74555	0.07816
-12	-1.58641	4.4278	0.05612	0.00035	0.00082	0.16056	0.78215
-11	-1.37263	3.8179	0.79210	0.00570	0.00662	0.07824	0.11734
-10	-1.22167	3.7828	0.01156	0.53788	0.44944	0.00033	0.00079
-9	-0.92823	3.5848	0.00037	0.44651	0.55201	0.00044	0.00068
-8	-0.73189	2.9503	0.82896	0.00477	0.00544	0.08911	0.07172
-7	-0.69520	3.6467	0.18199	0.00238	0.00184	0.76137	0.05242
-6	-0.66942	3.6542	0.05394	0.00039	0.00102	0.11559	0.82906
-5	-0.57678	2.9302	0.01362	0.55454	0.43087	0.00072	0.00025
-4	-0.39169	2.7326	0.01100	0.42165	0.56589	0.00092	0.00054
-3	-0.37529	2.1176	0.93572	0.00944	0.00708	0.01980	0.02796
-2	-0.22538	1.2663	0.94730	0.02153	0.01475	0.00719	0.00923
-1	-0.21034	2.1105	0.04245	0.56686	0.38888	0.00122	0.00059
1	-0.11444	1.9803	0.01875	0.32995	0.52482	0.09089	0.03558
2	-0.08536	2.7102	0.05273	0.00090	0.00197	0.20290	0.74150
3	-0.04464	0.8147	0.40563	0.37807	0.21577	0.00032	0.00021
4	-0.01224	0.8474	0.09115	0.33877	0.56988	0.00010	0.00009
5	-0.00104	0.4051	0.00904	0.75482	0.23612	0.00001	0.00001

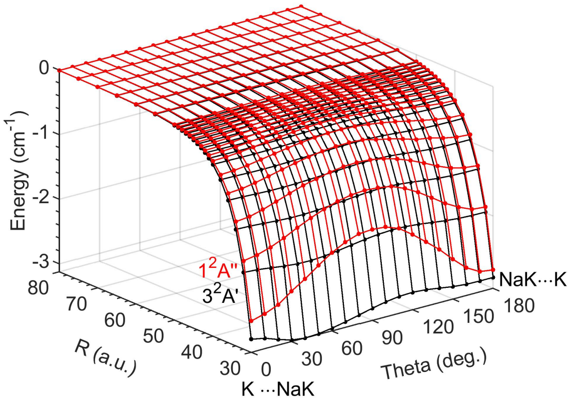

FIG. S2: Computed long-range PESs for the $3^2A'$ and $1^2A''$ excited states at $r = r_e(b) = 6.62$ a.u..

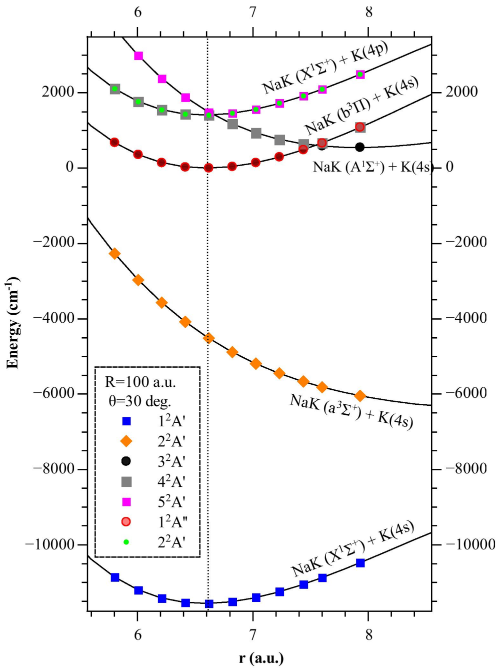

FIG. S3: Correlation diagram between the electronic states of the K···NaK trimer and those of the separated K atom and NaK dimer. The symbols depict one-dimensional cuts of the computed long-range PESs of K···NaK, in r coordinate in the interval [5.80 a.u.-7.93 a.u.], and $R = 100$ a.u., $\theta = 30°$, for the five lowest A' states and the two lowest A'' states. The zero of energies is fixed at the energy of NaK(b,$r = 6.62$ a.u.)+K(4s). The solid lines hold for the computed PECs of NaK in the lowest electronic states $X^1\Sigma^+$, $a^3\Sigma^+$, $b^3\Pi$, $A^1\Sigma^+$ (accompanied by a free K(4s) atom at infinity), as well as of the $X^1\Sigma^+$ PEC shifted by the excitation energy of K(4p). The vertical line marks the position of the minimum of the X and b PECs of NaK.

IV. EXAMPLES OF RADIAL WAVE FUNCTIONS OF THE K···NaK COMPLEX

We display in Fig. S4 the radial wave functions for some of the weakly bound levels of NaK ···K of A' symmetry corresponding to large computed PA rates, and on the same distance range, the energy-normalized radial wave function of the initial scattering state of PA with a collision energy $E = k_B \times 200$ nK. The spatial overlap between the initial state wave function and the final state wave functions of the proposed PA process is dominated by the value of the bound-state wave function at its first maximum from the right-hand side. For the chosen levels, a good overlap with the scattering wave function is indeed expected, as suggested by the vertical arrows, thus providing the main contribution to the computed PA rate.

V. PREDISSOCIATING RESONANCES

We assigned predissociating resonances, *i. e.* quasibound levels located between the NaK(b, $v_b = 0, j_b = 0$)+K(4s) and the NaK(b, $v_b = 0, j_b = 1$)+K(4s) limits, using the MFGH method and the stabilization method, as in Ref. 3. In brief, by extending the upper bound of the grid to sufficiently large distances $R_{\text{max}} = 1000$ a.u. to properly describe the continuum with a large number of artificial levels, we identified stable eigenvalues by plotting the quantity β, identical to B_n^J from Eq. 12 of the main text, as a function of energy (Fig. S5). It is not a rotational constant in a rigorous way, as the related eigenfunctions involve a continuum part, but it provides a convenient way to estimate the width of the resonances via the energy range on which the continuum is perturbed by the relevant bound level. We see that the first four resonances are quite narrow (≈ 0.001 cm^{-1}), and could probably be detected. Note that in these calculations, we set the off-diagonal terms of the potential energy matrix to zero for $R < R_{\text{sr}}$. We see in the figure that the results are almost unchanged if these off-diagonal terms are kept constant in the short-range.

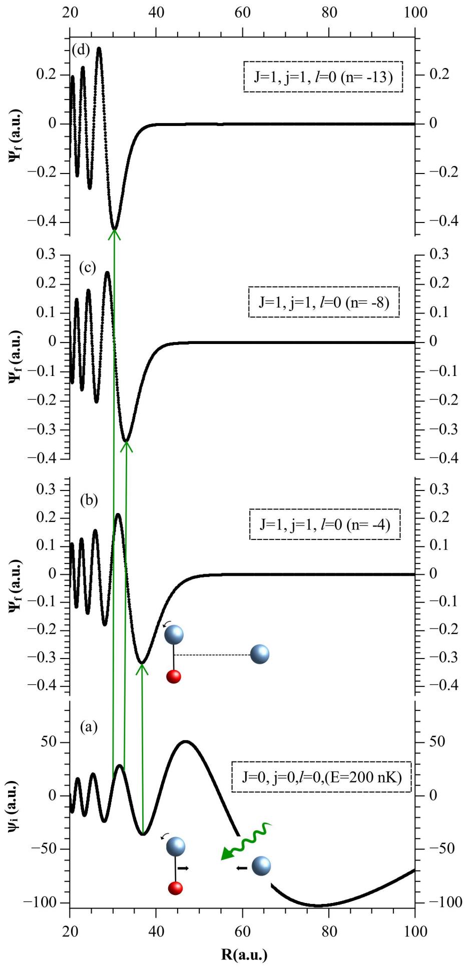

FIG. S4: (a) Energy-normalized radial wave function for the initial scattering state of PA, associated to the NaK(X, $v_X = 0, j_X = 0$)+K($4s$) threshold at a collision energy $E = k_B \times 200$ nK; (b)-(d) Radial wave functions of the final weakly-bound states of NaK $\cdots$ K associated with the energy levels $n = -4, -8, -13$ in Table III. The vertical arrows illustrate the good matching of the first oscillation (starting from the right-hand side) of the bound-state wave functions with the continuum wave function.

[1] Aymar, M.; Dulieu, O. Calculations of transition and permanent dipole moments of heteronuclear alkali dimers NaK, NaRb and NaCs. *Mol. Phys.* **2007**, *105*, 1733.

[2] Vexiau, R.; Borsalino, D.; Lepers, M.; Orbán, A.; Aymar, M.; Dulieu, O.; Bouloufa-Maafa, N. Dynamic dipole polarizabilities of heteronuclear alkali dimers: optical response, trapping and control of ultracold molecules. *Int. Rev. Phys. Chem.* **2017**, *36*, 709.

[3] Osséni, R.; Raoult, M.; Dulieu, O. Optimization of GMQDT reference functions for Feshbach resonance characterization. *J. Phys. B* **2009**, *42*, 185202.

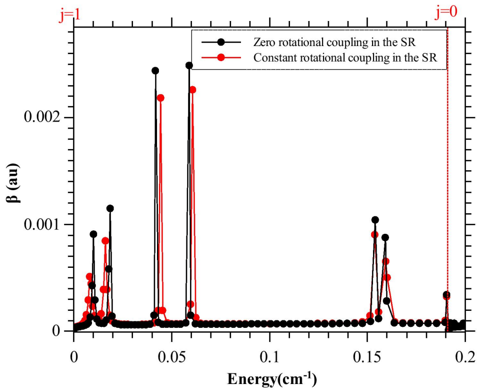

FIG. S5: Eigenvalues above the lowest dissociation limit, resulting from the MFGH solution of the coupled equations, after application of the stabilization approach. The predissociation resonances appear as isolated peaks between the asymptotic channels $j_b = 0, \ell = 1$ and $j_b = 1, \ell = 0$ in the excited state $3^2A'$ correlated to NaK(b,$v_b = 0$)+K($4s$). All the other dots correspond to the discretization of the continuum induced by the finite grid in R used in MFGH. Via the MFGH method, we assign to each eigenvalue a parameter β (calculated from Eq. 12 of the main text) analogous to the rotational constant for a physical level. We compare two cases for the off-diagonal terms of the potential energy matrix at short distances $R < R_{\rm sr}$: vanishing couplings (black circles), and constant couplings (red circles).

VI. Fitted asymptotic coefficients of the 1^2A'' state

This section contains the C_6 and C_8 coefficients (in a.u.) obtained from fitting the long-range PES of the 1^2A'' state of K-NaK correlated to the dissociation limit NaK(b^3Π) + K(4s) to the multipolar expansion:

V(R,r,theta)= -C_6/R^6 - C_8/R^8 + E_infinity(r)

****

r (a.u.)	E_infinity(r)[Hartree]
5.804	0.00307041
6.008	0.00161467
6.212	0.00065896
6.416	0.00014088
6.62	0.00000160
6.824	0.00018670
7.028	0.00064657
7.232	0.00133645
7.436	0.00221628

****

C_6(r,theta)[a.u.]
Column=C_6(r=5.804-7.436 with a step 0.204 a.u., theta) [a.u.]
Row=C_6(r, theta=0-180 with a step 10 deg.) [a.u.]

Theta	r=5.804	r=6.008	r=6.212	r=6.416	r=6.620	r=6.824	r=7.028	r=7.232	r=7.436
0	5708.690392	5670.992040	5805.222515	5980.394467	6095.999263	6190.305136	6257.740386	6342.205827	6735.316226
10	5581.583031	5642.144348	5765.372703	5875.195711	6047.072427	6127.286818	6164.850692	6245.013654	6622.007193
20	5381.557261	5483.191738	5544.634531	5730.765968	5873.857663	5978.851411	6094.789558	6123.328752	6333.002995
30	5123.378849	5207.922618	5297.880244	5516.517874	5665.317153	5755.560759	5832.160850	5956.541312	6110.918099
40	4854.549931	4952.565210	5000.244164	5173.659951	5358.254672	5450.315659	5540.005169	5628.318592	5803.274232
50	4585.692055	4637.571064	4759.374679	4901.047728	5085.954099	5193.472855	5211.846048	5329.611835	5516.118248
60	4374.558695	4414.852736	4502.560765	4636.200422	4796.487916	4900.503839	4967.291205	5008.728985	5209.680529
70	4240.272882	4232.210992	4311.473779	4443.800597	4544.451867	4634.190873	4750.653415	4810.941975	4958.098111
80	4116.193329	4123.923100	4209.440466	4347.522628	4441.550674	4541.248098	4621.597571	4670.282525	4856.185531
90	4102.508411	4168.249062	4236.466131	4328.066719	4495.341330	4510.634167	4665.425899	4675.886963	4832.321604

100	4234.017129	4298.655086	4408.323909	4506.699904	4676.644664	4714.292777	4795.593753	4876.797653	5011.881105
110	4496.447238	4501.852830	4639.830071	4749.854800	4908.077128	5017.176191	5067.235601	5139.003223	5349.661042
120	4835.339117	4900.993859	4978.910982	5100.510463	5247.862126	5391.741878	5476.416089	5541.263540	5765.634342
130	5300.278518	5359.466608	5433.937723	5565.483347	5719.189144	5831.880174	5957.595301	6014.129721	6233.312048
140	5908.654094	5955.503052	6043.590226	6197.474568	6295.098914	6397.484215	6460.460024	6548.313032	6741.879486
150	6576.637140	6602.569467	6698.364634	6798.607199	6904.201632	6976.732741	6990.514200	7072.185473	7200.795242
160	7172.816985	7211.316467	7266.017984	7349.414604	7427.063555	7473.955384	7466.043180	7496.401782	7645.964169
170	7586.460447	7617.931761	7666.167077	7749.151559	7789.476223	7767.282949	7795.407254	7788.816108	7944.106570
180	7746.855683	7765.968098	7816.425773	7887.371511	7927.458389	7898.974178	7919.907497	7891.959253	8074.683733

****

C_8(r,theta)[a.u.]

Column=C_8(r=5.804-7.436 with a step 0.204 a.u., theta) [a.u.]

Row=C_8(r, theta=0-180 with a step 10 deg.) [a.u.]

Theta	r=5.804	r=6.008	r=6.212	r=6.416	r=6.620	r=6.824	r=7.028	r=7.232	r=7.436
0	1927959.969	2228645.596	2431216.089	2625681.784	2885965.071	3181539.428	3538448.641	3886310.821	4068151.654
10	1883370.722	2100046.023	2301945.525	2534243.099	2744663.470	3056249.006	3405337.095	3763412.449	3951040.560
20	1720158.646	1892243.651	2112583.799	2263572.084	2465975.255	2711748.091	2968695.682	3325709.126	3584837.508
30	1533365.186	1684766.563	1850659.682	1943271.003	2101945.076	2315371.932	2552278.171	2768682.714	2992966.767
40	1327987.919	1434547.616	1618809.979	1705822.091	1797390.582	1963054.546	2141554.110	2329187.950	2476940.976
50	1176739.804	1286480.329	1360337.121	1434758.913	1486886.349	1602201.075	1807986.905	1930177.246	2019676.440
60	1024435.988	1116163.980	1188787.067	1232783.702	1274202.982	1357077.168	1473675.685	1610844.454	1643260.248
70	872832.2102	977755.0194	1035313.419	1064995.673	1124471.800	1194920.151	1249020.473	1345843.718	1387641.127
80	786332.2647	873548.0309	914477.5892	926367.4830	972230.5901	1018803.916	1083762.415	1170835.254	1169473.608
90	727130.5274	769786.3906	818735.7880	855157.6949	846753.2512	949116.3825	953218.8106	1063770.073	1074918.509
100	661458.3831	707031.3608	717782.0981	754753.1281	739652.4659	827260.3113	912559.6996	975128.6051	1021441.189
110	630407.9911	724930.4483	735843.7530	774634.6544	784395.5773	832150.7916	950786.8810	1032737.067	1025076.663
120	653430.6281	720099.8352	790897.1626	866691.1300	903433.4396	946659.4190	1037383.763	1148439.171	1166257.626
130	688873.5636	783724.0652	874365.2803	937337.6096	994460.2333	1093297.140	1184395.681	1333484.448	1381044.749
140	646123.2897	769641.6126	874622.2090	943189.1320	1073961.654	1203001.014	1371135.003	1533780.947	1648301.870
150	577626.7892	734467.8734	855434.7010	991843.4482	1135635.937	1313175.916	1547082.797	1749266.603	1966321.986
160	533203.9222	697140.5740	863435.9861	1035425.255	1213555.442	1430633.791	1706034.491	1996189.170	2233827.830
170	523871.3068	705432.9642	887910.0311	1070571.260	1286435.099	1565805.482	1830195.806	2190094.384	2415778.680
180	530790.6309	726136.5307	915786.0859	1103162.597	1327933.095	1616124.336	1890428.061	2251871.588	2461515.425

****

VII. Fitted asymptotic coefficients of the 3^2A'' state
This file contains the C_6 and C_8 coefficients (in a.u.) obtained from fitting the long-range PES of the 3^2A' state of K-NaK correlated to the dissociation limit NaK(b^3Π) + K(4s) to the multipolar expansion:

V(R,r,theta)= -C_6/R^6 - C_8/R^8 + E_infinity(r)

****

r (a.u.)	E_infinity(r)[Hartree]
5.804	0.00307034
6.008	0.00161460
6.212	0.00065890
6.416	0.00014083
6.62	0.00000156
6.824	0.00018666
7.028	0.00064654
7.232	0.00133643
7.436	0.00221622

****

C_6(r,theta)[a.u.]
Column=C_6(r=5.804-7.436 with a step 0.204 a.u., theta) [a.u.]
Row=C_6(r, theta=0-180 with a step 10 deg.) [a.u.]

Theta	r=5.804	r=6.008	r=6.212	r=6.416	r=6.620	r=6.824	r=7.028	r=7.232	r=7.436
0	6217.125687	6406.427228	6614.043500	6993.633319	7251.097403	7537.761026	7853.752236	8133.775433	8716.527001
10	6298.637273	6470.631963	6713.261316	7047.368309	7309.794806	7593.616375	7902.522344	8203.036448	8784.772530
20	6357.867482	6581.664359	6822.235888	7089.059278	7361.019006	7645.614174	7939.992726	8241.253596	8836.982188
30	6393.424035	6616.664890	6853.868212	7112.714178	7377.351613	7650.236078	7930.257274	8215.195033	8778.013017
40	6425.847052	6643.972353	6876.317367	7124.632805	7377.004890	7635.643716	7898.059437	8163.990197	8700.932744
50	6427.405967	6646.027864	6869.859542	7109.048626	7347.797729	7591.621422	7837.579917	8084.379884	8591.435875
60	6401.319087	6615.729853	6835.015294	7062.237666	7292.190114	7519.983551	7752.790328	7986.573086	8468.340112
70	6349.091704	6561.231786	6775.588768	6997.415738	7217.971437	7439.825938	7663.541370	7888.717860	8291.395365
80	6280.748313	6492.239826	6706.464157	6926.325745	7144.990718	7366.303483	7590.048681	7816.338653	8046.362592
90	6214.565902	6427.642900	6644.945227	6869.397674	7093.488069	7321.049323	7552.560225	7789.585777	8032.644429
100	6169.210008	6386.369110	6609.318171	6843.713788	7076.907208	7317.019866	7563.234995	7818.646037	8083.825032

110	6161.235336	6384.204443	6614.530289	6858.066743	7105.164736	7360.016200	7624.422759	7900.606809	8189.348563
120	6199.897515	6427.896917	6664.329529	6918.190783	7177.677700	7446.590669	7728.273830	8022.167743	8329.189444
130	6284.528043	6512.712769	6756.161695	7018.773229	7286.243549	7566.294383	7860.090744	8167.196100	8484.960008
140	6402.757760	6632.931049	6878.997202	7147.874010	7419.763578	7704.609852	8004.891837	8318.467254	8642.278826
150	6534.434229	6766.065154	7011.534303	7280.758081	7555.414885	7842.431481	8142.264307	8457.980496	8785.979629
160	6655.951404	6887.444941	7129.533762	7398.267195	7672.736917	7958.449780	8257.047185	8572.451528	8900.896147
170	6743.322889	6972.597502	7212.712436	7481.389466	7752.418022	8036.724334	8336.188100	8648.444404	8972.971487
180	6769.456230	6997.585820	7237.964494	7505.875067	7775.819239	8060.151531	8357.354729	8669.063313	8992.586265

****

C_8(r,theta)[a.u.]
Column=C_8(r=5.804-7.436 with a step 0.204 a.u., theta) [a.u.]
Row=C_8(r, theta=0-180 with a step 10 deg.) [a.u.]

Theta	r=5.804	r=6.008	r=6.212	r=6.416	r=6.620	r=6.824	r=7.028	r=7.232	r=7.436
0	1897706.235	2031752.501	2121372.023	2129603.700	2247985.410	2365931.463	2478300.357	2648729.692	2907562.180
10	1923459.533	2039153.275	2132482.167	2162527.839	2290681.552	2425459.847	2559005.623	2726114.865	2953037.871
20	1990010.220	2082116.995	2187581.492	2286908.736	2420553.160	2569965.665	2735864.135	2923586.432	3056581.797
30	2130049.862	2219474.699	2327132.842	2433579.350	2572713.767	2729936.374	2903814.611	3098596.747	3162145.271
40	2247298.606	2329251.568	2425854.489	2522508.468	2648762.037	2789640.909	2944662.549	3115555.134	3203953.088
50	2320187.613	2388722.803	2472487.376	2550815.041	2656566.539	2771636.421	2895544.769	3031505.295	3164954.338
60	2327111.740	2390109.057	2464382.834	2534221.772	2622782.270	2722223.986	2822629.044	2925688.708	3086379.495
70	2269052.023	2334359.579	2409658.136	2477400.583	2564227.389	2655631.354	2748888.191	2842451.861	2997692.161
80	2166183.504	2237552.604	2318448.459	2391022.806	2481691.438	2575360.087	2670964.400	2767460.915	2921613.541
90	2041546.823	2118451.165	2205817.556	2282248.899	2377669.968	2477148.721	2580134.711	2685476.585	2843786.173
100	1915412.734	1996774.050	2087641.216	2165600.698	2265518.436	2370428.741	2482276.887	2598624.896	2766022.170
110	1803385.178	1887015.001	1980045.195	2061296.910	2163373.405	2274665.627	2395133.113	2526026.725	2707952.635
120	1721289.443	1808233.049	1903183.337	1987017.315	2093271.090	2212006.233	2342427.098	2492804.689	2689485.365
130	1683309.451	1775226.610	1870076.990	1954025.728	2065469.099	2189651.021	2329077.555	2491900.024	2704932.381
140	1695475.465	1783667.732	1874627.501	1952931.253	2061359.638	2185095.419	2324882.580	2490550.146	2705557.272
150	1748465.327	1818976.743	1897840.209	1959834.338	2050779.803	2158196.684	2285530.525	2436457.778	2629009.925
160	1808566.038	1859658.940	1915902.294	1950237.820	2011735.145	2090529.201	2189002.136	2306168.602	2461783.756
170	1856418.443	1887910.829	1920180.278	1925768.579	1959857.075	2008689.109	2074520.141	2162389.565	2282554.435
180	1876204.852	1899664.212	1920881.456	1914671.791	1936908.260	1972384.310	2026525.461	2100876.314	2199121.943

****
